# Spinal cord evaluation in multiple sclerosis: clinical and radiological associations, present and future

**DOI:** 10.1093/braincomms/fcae395

**Published:** 2024-11-06

**Authors:** B Mark Keegan, Martina Absinta, Julien Cohen-Adad, Eoin P Flanagan, Roland G Henry, Eric C Klawiter, Shannon Kolind, Stephen Krieger, Cornelia Laule, John A Lincoln, Steven Messina, Jiwon Oh, Nico Papinutto, Seth Aaron Smith, Anthony Traboulsee

**Affiliations:** Department of Neurology, Mayo Clinic, Rochester, MN 55905, USA; Department of Neurology, Johns Hopkins University, Baltimore, MD 21218, USA; Institute of Biomedical Imaging, Polytechnique Montreal, Montreal, Canada H3T 1J4; Department of Neurology, Mayo Clinic, Rochester, MN 55905, USA; Department of Neurology, University of California San Francisco, San Francisco, CA 94143, USA; Department of Neurology, Harvard Medical School, Boston, MA 02115, USA; Division of Neurology, University of British Columbia, Vancouver, Canada V6T 2B5; Department of Neurology, Mount Sinai, New York City, NY 10029, USA; Division of Neurology, University of British Columbia, Vancouver, Canada V6T 2B5; McGovern Medical School, UTHealth, Houston, TX 77030, USA; Department of Neurology, Mayo Clinic, Rochester, MN 55905, USA; Division of Neurology, University of Toronto, Toronto, Canada M5B 1W8; Department of Neurology, University of California San Francisco, San Francisco, CA 94143, USA; Institute of Imaging Science, Vanderbilt University, Nashville, TN 37232, USA; Division of Neurology, University of British Columbia, Vancouver, Canada V6T 2B5

**Keywords:** multiple sclerosis, spinal cord, magnetic resonance imaging, atrophy, pathology

## Abstract

Spinal cord disease is important in most people with multiple sclerosis, but assessment remains less emphasized in patient care, basic and clinical research and therapeutic trials. The North American Imaging in Multiple Sclerosis Spinal Cord Interest Group was formed to determine and present the contemporary landscape of multiple sclerosis spinal cord evaluation, further existing and advanced spinal cord imaging techniques, and foster collaborative work. Important themes arose: (i) multiple sclerosis spinal cord lesions (differential diagnosis, association with clinical course); (ii) spinal cord radiological–pathological associations; (iii) ‘critical’ spinal cord lesions; (iv) multiple sclerosis topographical model; (v) spinal cord atrophy; and (vi) automated and special imaging techniques. Distinguishing multiple sclerosis from other myelopathic aetiology is increasingly refined by imaging and serological studies. Post-mortem spinal cord findings and MRI pathological correlative studies demonstrate MRI’s high sensitivity in detecting microstructural demyelination and axonal loss. Spinal leptomeninges include immune inflammatory infiltrates, some in B-cell lymphoid-like structures. ‘Critical’ demyelinating lesions along spinal cord corticospinal tracts are anatomically consistent with and may be disproportionately associated with motor progression. Multiple sclerosis topographical model implicates the spinal cord as an area where threshold impairment associates with multiple sclerosis disability. Progressive spinal cord atrophy and ‘silent’ multiple sclerosis progression may be emerging as an important multiple sclerosis prognostic biomarker. Manual atrophy assessment is complicated by rater bias, while automation (e.g. Spinal Cord Toolbox), and artificial intelligence may reduce this. Collaborative research by the North American Imaging in Multiple Sclerosis and similar groups with experts combining distinct strengths is key to advancing assessment and treatment of people with multiple sclerosis spinal cord disease.

## Teaser

Keegan *et al*. in the North American Imaging in Multiple Sclerosis review the crucial importance in spinal cord imaging in assessment of multiple sclerosis. The authors focus on the clinical relevance and novel techniques showing promise for imaging the actual pathology present in the multiple sclerosis spinal cord.

## Graphical Abstract

**Graphical Abstract:**
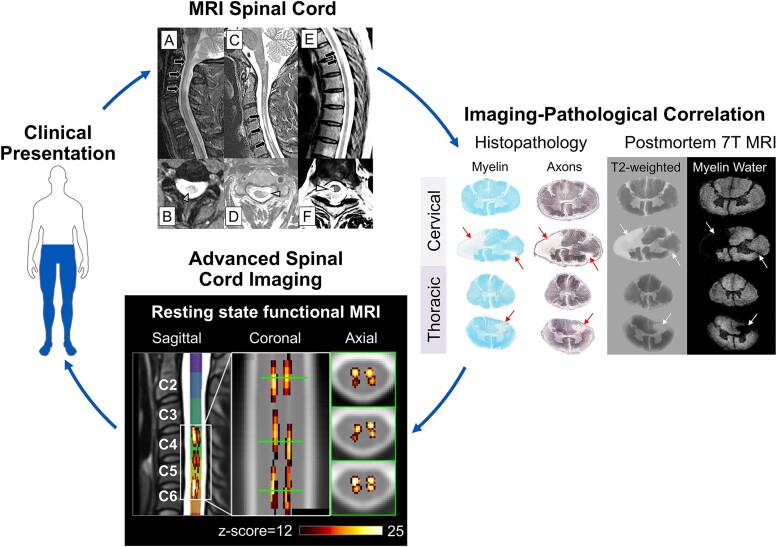

## Introduction

Demyelinating spinal cord disease is present in most people with multiple sclerosis. Assessment of multiple sclerosis spinal cord involvement clinically and radiologically remains less emphasized than multiple sclerosis brain assessment in patient care, basic and radiological research and therapeutic trials. It is likely that this focus on the brain rather than the spinal cord is from ongoing uncertainty of its clinical and therapeutic relevance, prior limited resolution of MRI spinal cord and substantial additional imaging costs. The North American Imaging in Multiple Sclerosis Spinal Cord Interest Group was formed to determine the contemporary landscape of multiple sclerosis spinal cord evaluation and foster collaborative efforts to further existing and advanced imaging techniques.

## MRI pathology of the spinal cord in multiple sclerosis

The spinal cord often has multifocal inflammatory demyelination in multiple sclerosis.^1^ Up to 90% of individuals within the multiple sclerosis spectrum have spinal cord demyelinated plaques detected by MRI (hereafter termed ‘lesions’).

**Figure 1:**
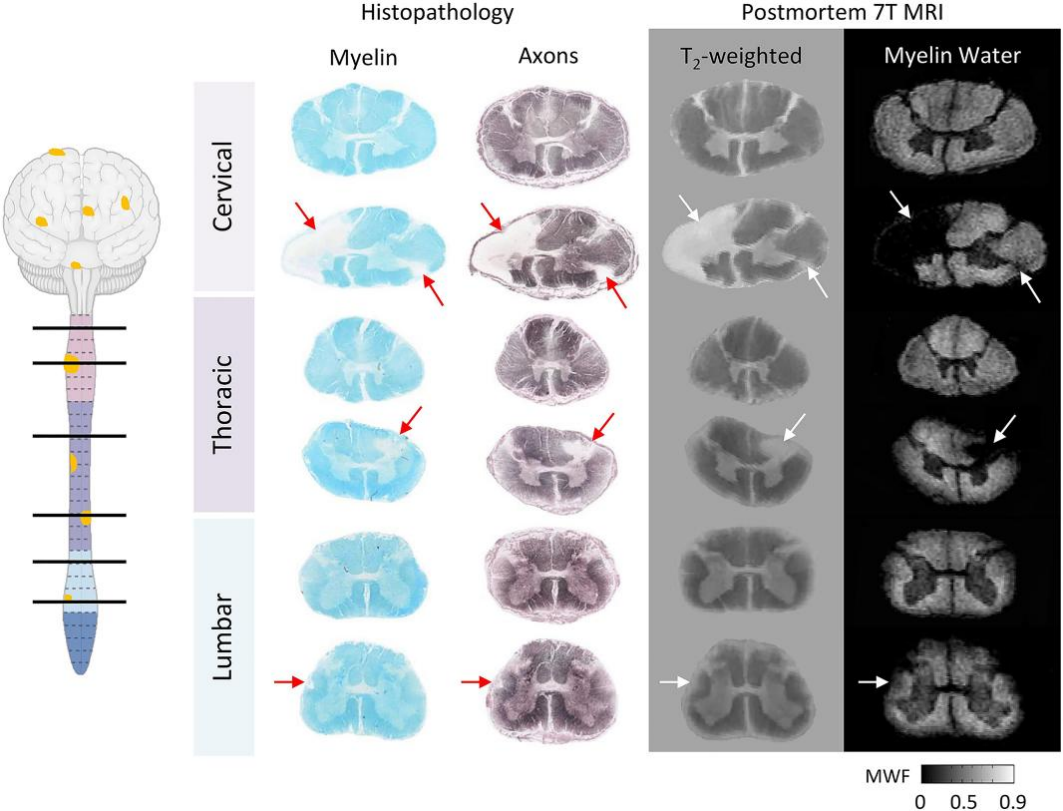
**Pathology and MRI of secondary progressive multiple sclerosis.** Histological staining and post-mortem 7 T MRI of a secondary progressive multiple sclerosis patient (female, 78 years old, 25-year disease duration). Focal lesions (arrows) are visible on cervical, thoracic and lumbar sections, highlighted by myelin (Luxol fast blue) and axon (Bielschowsky) staining, as well as on ex vivo 7 T T2-weighted and myelin water fraction MRI scans. Modified from Laule *et al.*^5^ with permission. MWF, myelin water fraction.

Multiple sclerosis spinal cord lesions involve the entire cord but are more frequently observed in cervical than thoracolumbar regions.^2,3^ Multiple sclerosis-specific lesions in the cord typically span over one to two vertebral segments, but rarely over three or more vertebral segments (a distinguishing feature of neuromyelitis optica spectrum disorder and myelin oligodendrocyte glycoprotein antibody-associated disease (Table 1; Supplementary Table 1). Spinal cord lesions are usually asymmetrical, more frequently involving dorsal and lateral columns versus anterior columns or central grey matter (GM). Spinal cord demyelination and axonal loss affect both white matter (WM) and GM,^4^ often without respecting GM–WM boundaries or occupying the entire cross-sectional cord area (Fig. 1).

**Table 1:** Differential diagnosis of myelopathies: timing of onset

Onset to nadir, *disease category*, specific diagnosis^a^
**Hyperacute (<12 h)**
*Vascular* (infarct)
*Functional neurologic disorder*
**Acute/subacute (12 h to 21 days)**
*Immune-mediated with demyelination*
AQP4+ neuromyelitis optica spectrum disorder
Myelin oligodendrocyte glycoprotein antibody-associated disease
Multiple sclerosis
*Infectious* (e.g., acute flaccid myelitis, schistosomiasis and West Nile Virus)
**Acute/subacute or progressive (12 h to months)**
*Immune-mediated other*
Paraneoplastic
Other neural antibody-associated
Rheum/connective tissue disease associated (e.g., Sjogren’s)
Sarcoid
**Progressive (21 days to many months)**
*Hereditary* (e.g., hereditary spastic paraplegia)
*Infectious* (e.g., HIV, human T-lymphotropic virus type 1 and syphilis)
*Inflammatory demyelinating* (e.g., primary progressive multiple sclerosis, progressive solitary sclerosis)
*Neurodegenerative* (e.g., amyotrophic lateral sclerosis)
*Structural* (e.g., spondylosis)
*Metabolic* (e.g., B12/N_2_O, copper and folate)
*Tumour* (primary, extrinsic or intramedullary metastases)
*Vascular* (spinal dAVF and cavernoma)

AQP4+ neuromyelitis optica spectrum disorder, aquaporin 4 antibody positive neuromyelitis optica spectrum disorder; dAVF, dural arteriovenous fistula; N_2_O, nitrous oxide.

^a^Time from onset to nadir (bold and underlined), disease category (italics), specific disease (regular text).

New multiple sclerosis lesions arise from inflammatory changes at the blood–brain/spinal cord barrier of post-capillary venules following radially oriented cord venous drainage,^6^ akin to perivenular brain lesions. Active MRI demyelinating lesions typically demonstrate nodular-enhancement or, less frequently, ring enhancement, with spinal cord oedema. After enhancement resolves, chronic cord lesions remain hyperintense on T2-weighted or short tau inversion recovery images (although decrease in size) and iso- or hypointense on conventional spin-echo T1-weighted sequences or heavily T1-weighted sequences such as T1-Magnetization-Prepared Rapid Acquisition Gradient Echo images or phase-sensitive inversion recovery (PSIR) images.

Chronic active spinal cord lesions are pathologically described in progressive multiple sclerosis, in many,^7-9^ but not all, studies.^10^ Unlike the brain,^11^ technical issues have limited the *in vivo* visualization of chronically inflamed spinal cord lesions using susceptibility-based MRI approaches. Recently, the cervical cord of both multiple sclerosis and non-neurologically affected subjects was imaged *in vivo* at 7 T MRI using an optimized susceptibility-weighted imaging protocol.^12^ Of relevance, this approach allowed the proper discrimination between the cord WM and GM, as well as the visualization of the central vein sign and paramagnetic rim lesions in about two-thirds of multiple sclerosis patients with spinal cord lesions.^12^

Similar to the brain, the spinal leptomeninges are enriched by immune inflammatory infiltrates, sometimes organized into B-cell lymphoid-like structures of proliferating B cells and follicular dendritic cells.^13^ Within spinal meninges, these lymphoid-like structures are described in a small proportion of progressive multiple sclerosis cases, in association with an outside-in gradient of subpial cord demyelination and axonal loss.^13^ In the brain, the blood–meningeal barrier disruption associated with meningeal inflammation has been visualized as leptomeningeal enhancement on delayed post-contrast T2-fluid attenuated inversion recovery images.^14^ Vercellino *et al*.^15^ have shown focal post-inflammatory leptomeningeal fibrosis as a potential pathological substrate that could explain persistent leptomeningeal enhancement. This imaging finding has not been reported in the literature for the spinal cord of multiple sclerosis patients.

Beyond focal demyelinated lesions, pronounced axonal, neuronal and especially synaptic loss likely results in overall spinal cord atrophy.^4,10,16^ Pathological findings are largely confirmed *in vivo* by advanced MRI methods highly sensitive to spinal cord microstructural alterations including demyelination and axonal loss.^10,16,17^

## Spinal cord lesions in multiple sclerosis diagnosis

MRI visible spinal cord lesions are included in dissemination in space and dissemination in time criteria of the revised McDonald diagnostic multiple sclerosis guidelines,^2^ as one of the four typical areas of central nervous system (CNS) involvement in multiple sclerosis. Primary progressive disease (primary progressive multiple sclerosis) in adults with isolated spinal cord involvement may be diagnosed with ≥2 spinal cord lesions with clinical progression ≥ 1 year and with cerebrospinal fluid (CSF) oligoclonal bands.

Radiologically isolated syndrome (RIS) leads to multiple sclerosis more commonly in those with spinal cord disease. A multivariable prospective analysis of 317 RIS patients found that persons < 37 years [hazard ratio = 4.04 (95% confidence interval = 2.00–8.15)], ≥1 spinal cord lesion [hazard ratio = 5.11 (1.99–13.13)] and baseline gadolinium-enhancing lesions [hazard ratio = 2.09 (1.13–3.87)] were independently associated with increased conversion to multiple sclerosis at 2 years.^18,19^

## Differential diagnosis of spinal cord lesions

**Figure 2:**
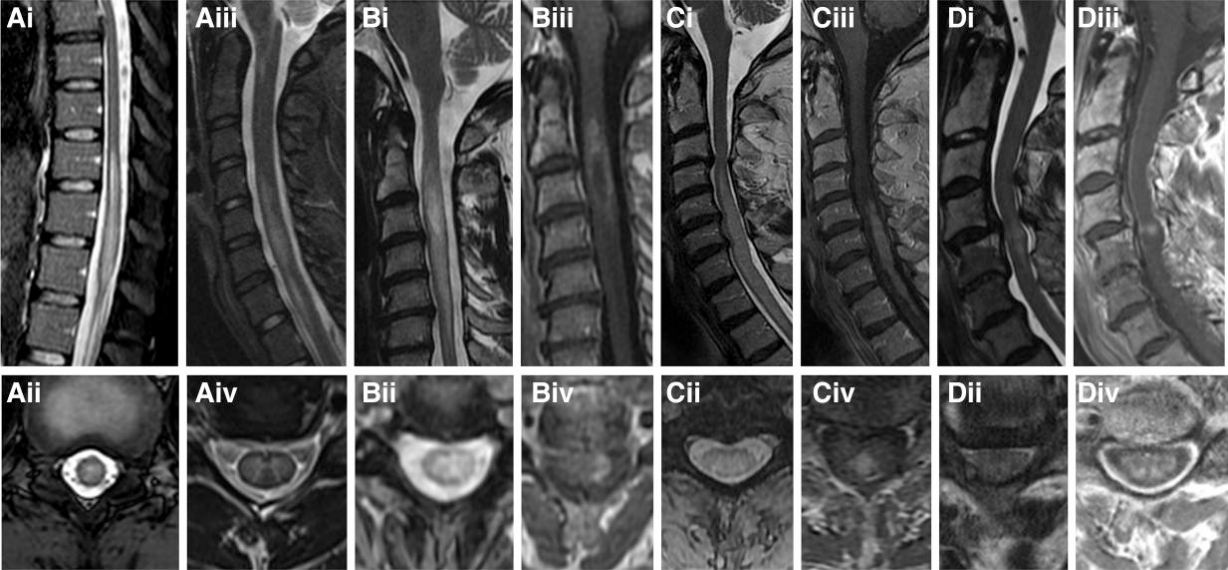
**MRI differential diagnosis of myelitis in multiple sclerosis.** (**A**) Myelin oligodendrocyte glycoprotein antibody-associated disease. (**B**) Aquaporin 4-IgG positive neuromyelitis optica spectrum disorder (AQP4+ neuromyelitis optica spectrum disorder). (**C**) Spinal cord sarcoidosis. (**D**) Cervical spondylotic myelopathy. Thoracic spine MRI reveals a sagittal T2 lesion in the lower thoracic spine involving the conus and extending <3 vertebral segments (**Ai**) with axial T2 images revealing a central T2 lesion (**Aii**) in a patient with myelin oligodendrocyte glycoprotein antibody-associated disease. Cervical spine sagittal MRI reveals a longitudinally extensive T2 lesion extending >3 vertebral segments (**Aiii**) with T2 hyperintensity restricted to the central GM on axial T2 images forming a H sign (**Aiv**, arrow) in a patient with myelin oligodendrocyte glycoprotein antibody-associated disease. Cervical spine sagittal MRI reveals a longitudinally extensive T2 lesion extending >3 vertebral segments (**Bi**) with central T2 lesion on axial images (**Bii**) with an elongated ring of enhancement on T1-weighted images post-gadolinium (**Biii**) with an open ring on axial T1-weighted images post-gadolinium (**Biv**) in a patient with AQP4+ neuromyelitis optica spectrum disorder. Cervical spine sagittal MRI reveals a longitudinally extensive T2 lesion extending >3 vertebral segments (**Ci**) with central T2 lesion on axial images (**Cii**) accompanied by linear dorsal subpial enhancement on sagittal and axial T1-weighted images post-gadolinium (**Ciii**, **Civ**) in spinal cord sarcoidosis. Cervical spine sagittal MRI reveals a T2 lesion extending <3 vertebral segments (**Di**) with axial T2 images showing some T2 hyperintensity (**Dii**) and evidence of a transverse band or ‘pancake-like’ enhancement with the width greater than the height (**Diii**) circumferentially involving the WM (**Div**).

A broad differential diagnosis of myelopathies is stratified by symptom onset speed (hyperacute, acute/subacute and chronic) (Table 1) and clinical and MRI clues (Table 1; Supplementary Table 1; Fig. 2).^20,21^ MRI should include two complimentary sagittal sequences T2, proton density; proton dense, short tau inversion recovery and axial T2 images, with gadolinium contrast at onset in all patients and gadolinium contrast, can be considered for follow-up images when clinically indicated gadolinium contrast when clinically indicated and PSIR as optional.^22^ Diffusion-weighted imaging is useful when evaluating hyperacute, severe myelopathies to assess for spinal cord infarction although has greater technical challenges than in the brain that can impact image quality. Initial spine MRI assesses for extrinsic compression (spondylotic, metastases and epidural abscess), including intradural extramedullary tumours (e.g. meningioma) and extradural compressive lesions (e.g. epidural abscess, spondylosis or metastases) After this, the MRI should be reviewed then for intrinsic spinal cord T2 lesions.^22^ A subacute myelopathy without MRI spinal cord T2 hyperintensity can suggest stiff-person syndrome or, if progressive, a genetic or degenerative aetiology.^22,23^ A normal MRI at clinical onset with subsequent intrinsic cord T2 hyperintensity may indicate spinal cord infarction or myelin oligodendrocyte glycoprotein antibody-associated disease.^24,25^ T2 lesion length and axial location are crucial to narrow the differential diagnosis of myelopathies (Table 1; Supplementary Table 1). Longitudinally extensive T2 lesions extending ≥3 vertebral segments are extremely rare in multiple sclerosis and more typical of aquaporin 4 antibody positive (AQP4+) neuromyelitis optica spectrum disorder or myelin oligodendrocyte glycoprotein antibody-associated disease and other non-multiple sclerosis myelopathies (Fig. 2).^26-28^ Occasionally, multiple short spinal cord T2 lesions can coalesce and artefactually appear longitudinally extensive on sagittal images, but axial images can help identify multiple separate short T2 lesions.^26^ Central axial lesions are more typical of myelin oligodendrocyte glycoprotein antibody-associated disease, AQP4+ neuromyelitis optica spectrum disorder and spinal cord sarcoidosis while axial T2 lesions restricted to the GM forming a ‘H sign’ is quite suggestive of myelin oligodendrocyte glycoprotein antibody-associated disease but can also occur in acute flaccid myelitis (Fig. 2).^29,30^ Central axial lesions are more typical of alternative aetiologies such as the ‘H sign’ in myelin oligodendrocyte glycoprotein antibody-associated disease.^31^ The presence of gadolinium enhancement and its pattern provide useful clues to the underlying aetiology, and these are summarized in Supplementary Table 1 and have been reviewed in detail previously, and some examples are shown in Fig. 2.^27,31^ Brain MRI can indicate multiple sclerosis or other inflammatory demyelinating disorders (i.e. AQP4+ neuromyelitis optica spectrum disorder and myelin oligodendrocyte glycoprotein antibody-associated disease). Notably, up to 10% of patients with cerebral myelin oligodendrocyte glycoprotein antibody-associated disease attacks may have an initial normal MRI highlighting the potential for radiologic lag in myelin oligodendrocyte glycoprotein antibody-associated disease and repeat MRI within a few weeks may reveal evolving T2-signal abnormalities.^32^ Moreover, myelin oligodendrocyte glycoprotein antibody-associated disease T2 lesions are very dynamic, frequently appearing and occasionally disappearing within the same attack differing from multiple sclerosis and AQP4+ neuromyelitis optica spectrum disorder in which the lesion number within the attack tends to be stable.^32^ Resolution of MRI T2 lesions over time is more common in myelin oligodendrocyte glycoprotein antibody-associated disease (80%) than in multiple sclerosis and AQP4+ neuromyelitis optica spectrum disorder (<10%), while new asymptomatic cord lesions are more typical of multiple sclerosis than myelin oligodendrocyte glycoprotein antibody-associated disease or AQP4+ neuromyelitis optica spectrum disorder.^33^ Focal lesional atrophy develops in multiple sclerosis, but longer segments of atrophy may suggest AQP4+ neuromyelitis optica spectrum disorder.^31,33^ Persistent spinal cord enhancement beyond 3 months argues against multiple sclerosis.

## Contribution to multiple sclerosis clinical course and prognosis

Spinal cord lesions occur across the multiple sclerosis disease spectrum,^34,35^ more frequently in progressive than relapsing-remitting multiple sclerosis,^36^ in 30–70% of people with RIS and in 30–70% of people with RIS, with higher numbers seen when PSIR cervical spinal cord imaging is performed versus conventional sagittal T2-based sequences.^37^

Despite the diagnostic role of spinal cord lesions, the utility of routine spinal cord monitoring is unclear with studies showing asymptomatic cord lesions in 12–15% people with multiple sclerosis and only 6% of scans with new T2 lesion only in the spinal cord.^38,39^ One study showed that new cervical spinal cord lesions in clinically stable people with multiple sclerosis was <2% on routine 3 T MRI spine over a very short follow-up not offering a benefit for routine monitoring.^40^ A retrospective study showed one-third of people had new T2 demyelinating lesions exclusively within the spinal cord and were asymptomatic, but gadolinium-enhancing lesions only within the spinal cord in 16% of patients monitored arguing strongly for spinal cord MRI assessment given the association with disability accrual.^41^ Thus, given the variances in the quality of spinal cord image acquisition and, therefore, reliability and accuracy in detecting new spinal cord lesions, there is a need for technological improvements in spinal cord imaging.

The presence of new spinal cord lesions may influence therapeutic decision-making by increasing the likelihood to transition to higher efficacy disease-modifying therapy.^42^ One study showed fully 25% of people with relapsing-remitting multiple sclerosis developed at least one new asymptomatic spinal cord lesion over a median follow-up of 17 months and those with either new asymptomatic spinal cord or brain lesions were more likely to experience a further clinical relapse.^43^ A single-centre retrospective study of 115 multiple sclerosis patients treated with first-line disease-modifying therapies analysed the presence of no evidence of disease activity with or without spinal cord imaging. New spinal cord lesions occurred in 13% of patients and including spinal cord imaging with routine brain MRI reduced short-term no evidence of disease activity 3 [absence of relapses, new lesions and expanded disability status scale (EDSS) change] by nearly 8% compared with brain MRI alone.^44^

While the role of routine spinal cord monitoring in multiple sclerosis is unclear,^22^ evidence that cord damage is important in predicting disability is more robust. The presence of spinal cord lesions is predictive of developing clinically definite multiple sclerosis in RIS and may have the greatest prognostic value of all clinical and radiological measures.^19,45^ Spinal cord lesions in clinically isolated syndrome (CIS) and early relapsing-remitting multiple sclerosis are associated with a higher risk of relapse, increased disability and conversion to secondary progression multiple sclerosis.^35,46^ Retrospective analysis of 207 patients found the presence of a spinal cord lesion in non-myelitis CIS increased risk of EDSS > 3.0 at 2 years (adjusted hazard ratio = 29.8, 95% confidence interval = 1.1–786.5, *P* = 0.042).^46^ In a study of 178 CIS patients, gadolinium-enhancing (odds ratio = 3.16, *P* < 0.01) or spinal cord lesions (odds ratio = 4.71, *P* < 0.01) on baseline MRI, performed within 12 weeks of clinical onset, independently predicted secondary progressive multiple sclerosis at 15 years.^35^ New spinal cord lesions over 1–3 years were associated with an increased risk of secondary progressive multiple sclerosis.^35^

Relationships between spinal cord atrophy and clinical progression are documented in all multiple sclerosis phenotypes,^31,47,48^ and tracking spinal cord atrophy over time provides substantial prognostic value.^49,50^ Casserly *et al*.^51^ performed a pooled analysis of 73 MRI studies. Smaller spinal cord cross-sectional area (CSA), typically measured at C2–3, was reported in progressive multiple sclerosis (primary progressive multiple sclerosis/secondary progressive multiple sclerosis) compared with relapsing-remitting multiple sclerosis/CIS and healthy controls. Pooled annual atrophy rates were −1.78%/year for all multiple sclerosis types and −2.08%/year for progressive multiple sclerosis.^51^ Moderate correlations are noted between spinal cord CSA and EDSS,^51,52^ and spinal cord atrophy also contributes to non-relapsing smoldering disease associated with clinical progression. A 12-year longitudinal study reported that silent progression and conversion to secondary progressive multiple sclerosis are predominantly related to early cervical cord atrophy (−2.19%/year).^47^ Both WM and GM spinal cord tissues are affected by atrophy, but GM atrophy is believed to start earlier and have the strongest correlation with disability.^36,53,54^ Given the unmet need to treat progressive multiple sclerosis, biomarkers predicting transition to progressive multiple sclerosis are crucially needed for evaluating treatment efficacy in clinical trials. By evaluating the degree of atrophy and its correlation with clinical outcomes, more effective treatment strategies can be developed, ultimately improving patients’ quality of life and prognosis.

Studies of spinal cord injury and long-term disability in pediatric onset multiple sclerosis are based on smaller cohorts with mixed results.^18,55^ A study of 20 pediatric onset multiple sclerosis compared with matched healthy controls showed no differences in CSA,^18,55^ while a study of 125 pediatric onset multiple sclerosis showed that the number of baseline cervical cord lesions was a moderate predictor (*β* = 0.22, *P* = 0.05) of higher EDSS 9 years later.^18^

## ‘Critical’ demyelinating lesions associated with progressive motor impairment

**Figure 3:**
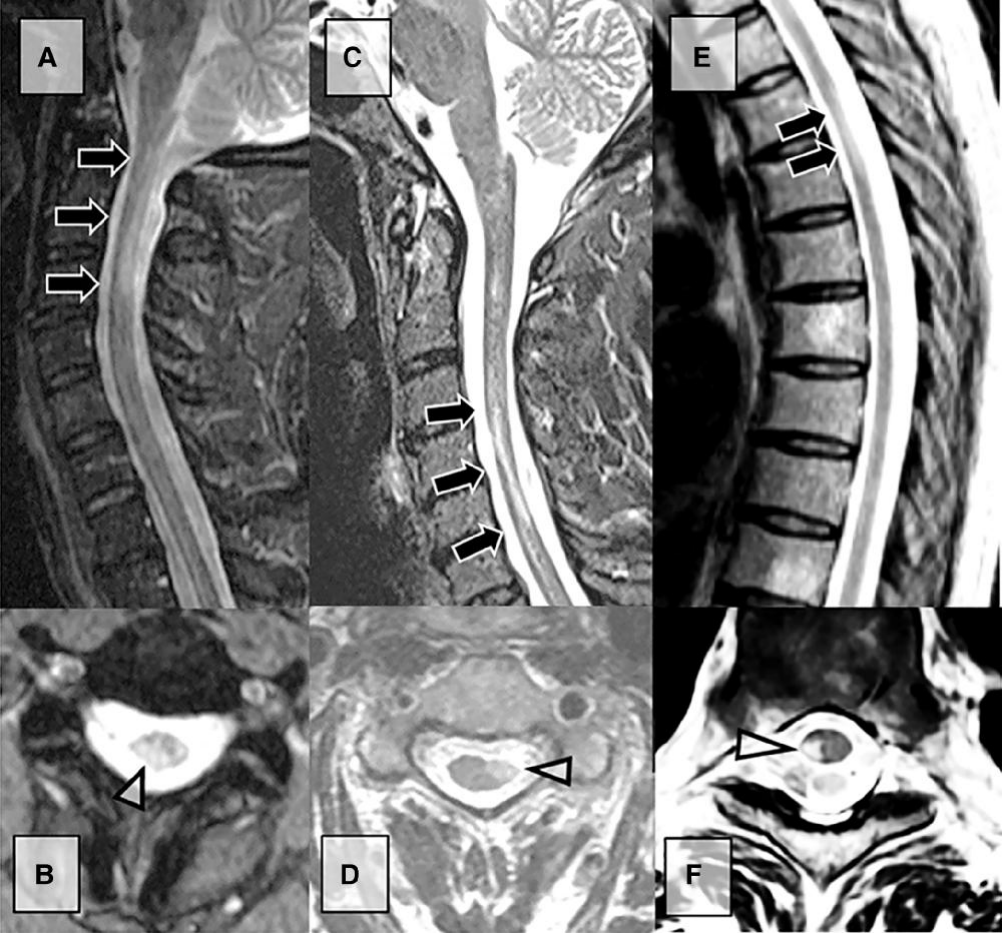
**Critical demyelinating lesions of the spinal cord.** ‘Critical’ demyelinating lesions of the spinal cord are anatomically associated with progressive motor impairment in people with multiple sclerosis; they are typically laterally based in the corticospinal tract and have focal spinal cord atrophy. MRI cervical spine sagittal (**A**) and axial (**B**) images showing ‘critical’ demyelinating lesion with T2 signal abnormality in the right lateral-dorsal cord with focal atrophy (black arrows and arrowhead) in a 53-year-old man with a 5-year history of progressive right upper motor neuron face-sparing hemiparesis. CSF showed four unique oligoclonal bands. MRI cervical spine sagittal (**C**) and axial (**D**) images showing ‘critical’ demyelinating lesion with T2 signal abnormality in the left lateral cord (arrows) with focal atrophy (open arrowhead) in a 59-year-old woman with a prior history of relapsing-remitting multiple sclerosis and an 8-year history of progressive left face-sparing upper motor neuron hemiparesis. Additional demyelinating lesion was seen in the lower thoracic spine with no volume loss (not shown). MRI thoracic spine sagittal (**E**) and axial (**F**) images showing ‘critical’ demyelinating lesion with T2 signal abnormality right hemispinal cord (arrows and open arrowhead) in a 58-year-old man with multiple sclerosis and progressive right lower extremity monoparesis.

Most people with progressive multiple sclerosis have progressive, asymmetrical myelopathic motor impairment potentially explained by multifocal demyelinating spinal cord lesions. While some people with spinal cord multiple sclerosis have diffuse proton dense signal abnormalities especially in chronic multiple sclerosis and such diffuse signal abnormalities may be a poor prognostic indicator in relapsing-remitting multiple sclerosis,^56,57^ most are seen as focal, short segment lesions often with isolated tract involvement.^58^ The specific contribution of any individual demyelinating lesion is usually uncertain as most people have innumerable focal cord lesions, making a clear association between progression and one or more specific demyelinating lesions challenging. Highly selected cohorts of people where a specific ‘critical’ demyelinating lesion can be anatomically associated with progressive motor impairment have been described (Fig. 3): (i) ‘progressive solitary sclerosis’ (one CNS demyelinating lesion anatomically consistent with progressive motor impairment)^59^; (ii) ‘progressive paucisclerosis’ (highly restricted, two to five total CNS lesions, with one ‘critical’ demyelinating lesion anatomically associated with progressive motor impairment)^60^; and (iii) ‘progressive, exclusively unilateral, hemi- or mono-paresis’ (a ‘critical’ demyelinating lesion with >5 CNS lesions).^61^ ‘Critical’ demyelinating lesions are typically in the spinal cord but occasionally involve supratentorial corticospinal WM tracts, brainstem or cervicomedullary junction.^60-62^ ‘Critical’ demyelinating lesions are characterized by moderate to severe focal atrophy, lateral column axial location and larger size than ‘non-critical’ spinal cord lesions.^63^ Age of onset of motor progression is similar in cohorts with ‘critical’ demyelinating lesions as in typical multiple sclerosis, and new inflammatory multiple sclerosis activity is uncommon in these cohorts. Those with long-term multiple sclerosis (>25 years) without spinal cord ‘critical’ type lesions maintain a relapsing multiple sclerosis course without progression,^64^ and people with tumefactive multiple sclerosis (mass-like, large demyelinating brain lesions) are unlikely to have progressive motor impairment without corticospinal tract spinal cord lesions.^64,65^

## Topographical model of multiple sclerosis and disease course

An emerging view of multiple sclerosis disease course is to conceptualize it as a continuum of relapsing and progressive mechanisms, and that loss of CNS reserve is the principal driver of long-term disability accumulation.^66^ It is hypothesized that the accelerated loss of brain and spinal cord volume depletes the compensatory mechanisms that constitute neurological reserve and that progression may become clinically apparent as reserve is lost. The topographical model of multiple sclerosis provides a unified visualization of multiple sclerosis clinical course across the spectrum of relapsing and progressive forms of the disease.^67^

The topographical model prioritizes lesion localization as the crucial factor in explicating multiple sclerosis clinical heterogeneity and derives clinical course by depicting the specific relationship between subclinical lesion burden and variable amounts of reserve intrinsic to different CNS regions.^68^ In this framework, the CNS is visualized as a pool of reserve with increasing depth, with the spinal cord and optic nerves at the shallow end, and the cerebral hemispheres comprising the deep end. To depict disease activity, lesions rise as focal peaks emerge from the base of the pool; lesions that cross the clinical threshold cause demonstrable symptoms of a multiple sclerosis relapse. In this model, spinal cord lesions—that shallow pool of reserve—are more likely to yield acute relapses of pyramidal, sensory and bowel/bladder symptoms and lead to long-term disability.^46^

The topographical model posits a distinct intersection between disease topography and loss of functional reserve driving multiple sclerosis clinical course: as time passes and functional reserve declines, progression clinically recapitulates the prior relapse symptoms, incrementally manifesting above the clinical threshold of a patient’s underlying disease topography.^67^ Thus, cord lesions are particularly apt to re-emerge insidiously as functional and structural resilience is lost, yielding the multifocal myelopathic symptoms as cardinal manifestations of multiple sclerosis disability progression.

This model draws from an array of empirical work summarized in this review, principally that the presence and number of spinal cord lesions predict a progressive clinical course, including the role of a singular critically located^64^ upper cervical cord lesion.^60^ That the specific clinical features of a patient’s disability progression could be topographically mapped to the cervical cord lesion location enhances the ability to model and predict disease course. Associations between spinal cord volume^47^ and multiple sclerosis outcomes further support the topographical model framework, as spinal cord atrophy also correlates with disability accumulation, consistent with a decline of spinal cord reserve as more recently defined by Sastre-Garriga *et al*.^69^ Focusing on advanced and emerging techniques is imperative for improving multiple sclerosis spinal cord imaging, as this most prognostically important localization is where our imaging is currently least sensitive and where the signal-to-noise ratio is in great need of optimization.^68^

## Quantitative analysis with conventional MRI

### Multiple sclerosis lesion segmentation

Given the clinical relevance of spinal cord lesions, their segmentation and quantification have been attempted for the prediction of disability and clinical progression. The crucial importance of both sagittal and axial imaging for spinal cord lesion segmentation is evident in that the detection rate is higher (up to 22%) compared with sagittal images alone.^70^ Manual segmentation of lesions is time-consuming and suffers from intra-/inter-rater variability,^71^ motivating the development of robust and automatic tools. Automatic lesion segmentation, including methods based on deep learning, has been thoroughly investigated over the past two decades for brain^72-74^ and spinal cord data^75^; however, the published methods are not optimally reliable.^72^ As multiple sclerosis lesions are small relative to the structure and various challenges discussed above need to be met are of interest (spinal cord), algorithms suffer from high class imbalance leading to poor performance. This issue can be mitigated by segmenting lesions in a template space (i.e. PAM50 template),^76^ allowing one to restrict the spatial domain where lesions are likely to appear. Another challenge is that inter-rater variability in ground truth labels creates a bottleneck in the performance of automatic segmentation methods^77^; however, modelling rater bias is a viable solution.^77^ A third challenge in artificial intelligence is that deep segmentation models have a hard time generalizing to images acquired with different parameters, which is common in multi-centre studies. To boost deep learning model generalization, one could aggregate data from multiple hospitals, but legal constraints lead to difficulty in sharing medical data between institutions. Federated learning can overcome this issue,^78^ where each hospital trains a model using their own data and model weights are then shared in lieu of imaging data. Model parameters are aggregated and sent back to each hospital for use and further training, recently demonstrated for glioblastoma segmentation.^79^ Federated learning may not be a panacea however and challenges remain.^80^

## Spinal cord atrophy

### How to compute atrophy

Introduced by Losseff *et al*. in 1996, spinal cord atrophy is commonly assessed by calculating CSA of the spinal cord on a segmented axial MRI image (Table 2).^89^ While assessment of spinal cord atrophy is informative for understanding multiple sclerosis and related disability progression, it has yet to be used routinely in clinical assessments or in clinical trials. CSA accuracy is mainly limited by image resolution, so acquiring data with high axial resolution and averaging CSA across multiple slices (i.e. C2–3 vertebral levels) increases precision. Manual spinal cord CSA and GM segmentation is hampered by rater bias, and various techniques with differing levels of automation have been developed over the past 30 years,^90^ available in software packages,^91^ like JIM^88^ (https://www.xinapse.com/j-im-9-software/) and Spinal Cord Toolbox (https://spinalcordtoolbox.com/).^90,92^

**Table 2:** Published manuscripts (from 2018) that include sample size/power calculation estimates of subjects that would be required to use cervical spinal cord area/volume as end-point in a clinical trial testing multiple sclerosis therapies

Manuscript	Type of study cohort/data used	MRI protocol and measurement method	Results reported for a longitudinal scenario
Cawley *et al*.^81^	Longitudinal, single-centre, Philips 3 T 44 progressive multiple sclerosis (26 primary progressive multiple sclerosis/18 secondary progressive multiple sclerosis) 29 healthy participants. Power based on actual 1 year % changes measured on the cohort	CSA measured at C2–3 from axial 3D T1w spinal cord images using active surface model (JIM)	80% power, 0.05 statistical significance. One-year neuroprotective clinical trial in progressive multiple sclerosis. 50% treatment effect: 57 patients for primary progressive multiple sclerosis, 546 for secondary progressive multiple sclerosis (146 for the entire progressive multiple sclerosis cohort) per arm. 30% treatment effect: 157 patients for primary progressive multiple sclerosis, 1538 for secondary progressive multiple sclerosis (401 for progressive multiple sclerosis) per arm
Moccia *et al*.^82^	Longitudinal, multi-centre, vendor, field: 282 multiple sclerosis patients (52 CIS, 196 relapsing-remitting multiple sclerosis, 34 progressive multiple sclerosis) and 82 healthy participants. Power calculations based on the actual 1 year % changes measured on the cohort	CSA measured with SCT from C2–5 on sagittal 3D T1w spinal cord acquisitions. Generalized boundary shift integral (GBSI) used to measure % changes.	80% power, 0.05 statistical significance. Hypothetical clinical trial over a 1-year period. 60% treatment effect: CIS: 106 versus 830, relapsing-remitting multiple sclerosis: 95 versus 335, progressive multiple sclerosis: 44 versus 215 patients per arm for GBSI and SCT, respectively. 30% treatment effect: CIS: 419 versus 3314, relapsing-remitting multiple sclerosis: 374 versus 1336, progressive multiple sclerosis: 170 versus 854 patients per arm for GBSI and SCT, respectively
Tsagkas *et al*.^83^	Longitudinal, single-centre, Siemens 1.5 T. 60 multiple sclerosis patients (12 primary progressive multiple sclerosis, 24 relapsing-remitting multiple sclerosis and 24 secondary progressive multiple sclerosis) were selected retrospectively. MRI data of 6 years of follow-up (7 annual time points) used	SCV measured with CORDIAL over a 35-mm long SC segment, starting 27 mm below the cisterna points on 3D T1w sagittal brain acquisitions	95% power, 0.05 statistical significance. 17.5% effect as a meaningful atrophy reduction, as observed in the brain in a clinical trial investigating the therapeutic effect of a treatment compared to placebo^84^; 94 patients for 1 year, 59 patients for 2-year follow-up per arm
Moccia *et al*.^85^	Longitudinal, Phase 2 clinical trial, 1.5 and 3 T scanners of multiple vendors; 114/169 successful primary progressive multiple sclerosis with cord images. Baseline and Week 48 MRI scans used.	CSA measured with SCT from C2–5 on sagittal 3D T1w spinal cord acquisitions. Generalized boundary shift integral (GBSI).	80% power, 0.05 statistical significance. Hypothetical clinical trial evaluating a neuroprotective medication over a 1-year period. 60% treatment effect: 257 versus 395 patients per arm for GBSI and SCT, respectively; 30% treatment effect: 1027 versus 1581 patients per arm for GBSI and SCT, respectively
Papinutto *et al*.^86^	Cross-sectional, single-centre, Siemens 3 T 129 healthy participants plus previous cohort of 113 multiple sclerosis with same protocol used for cross-sectional measures approach to longitudinal power. Estimates and data from Casserly *et al*.^51^ were used for the longitudinal scenario estimates	CSA measured with JIM from single-slice 2D T1w PSIR at C2-C3 and normalized with v-scale and axial canal product	90% power, 0.05 statistical significance. Longitudinal: 1.78% pooled annual cord atrophy for multiple sclerosis patients reported in Casserly *et al*.^51^ used for this estimate, together with a group standard deviation of annual CSA reduction of 0.48% (from healthy participants, assuming a linear trend). 30% treatment effect: 36 patients per arm. Cross-sectional: 7.77 mm^2^ (∼10%) CSA difference between relapsing-remitting multiple sclerosis and progressive multiple sclerosis patients was previously detected using the same 2D PSIR MRI protocol. To detect relapsing-remitting multiple sclerosis/progressive multiple sclerosis differences: 30 patients per arm
Bautin and Cohen-Adad^87^	Cross-sectional, multi-centre, vendor, field Atrophy and patient repositioning simulations on an open-access data set of 260 healthy participants and data from Casserly *et al*.^51^ used to estimate % atrophy commonly seen in multiple sclerosis	Average CSA measured with SCT between vertebral levels C3 and C5 (included) on sagittal 3D T1w and T2w images	Longitudinal atrophy (between paired study arms) of 0.8%. 60 ± 25.1 per arm using T1w images. 10 ± 1.2 per arm using T2w images. Cross-sectional: Atrophy of 2% between unpaired study arms. 467 ± 13.9 per arm using T1w images. 467 ± 3.2 per arm using T2w images
Valsasina *et al*.^88^	Longitudinal, multi-centre, vendor, field Cohort I: 8 HC and 28 multiple sclerosis patients (10 relapsing-remitting multiple sclerosis/18 progressive multiple sclerosis)—one site Siemens 1.5 T Cohort II: 25 HC and 63 multiple sclerosis patients (54 relapsing-remitting multiple sclerosis/9 progressive multiple sclerosis)—three sites—3 T scanners of multiple vendors	Average CSA between C1/C2 and C5 (1.5 T) or C7 (3 T) on 3D T1w spinal cord images using SCT’s PropSeg registration-based approach included in JIM 9	90% power, 0.05 statistical significance. Treatment effect in a hypothetical placebo-controlled randomized clinical trial aimed at reducing atrophy over 1-year. 50% treatment effect: SCT: 77/951 per arm (Cohort I/Cohort II). JIM 9: 52/348 per arm (Cohort I/Cohort II). 30% treatment effect: SCT: 211/2640 per arm (Cohort I/Cohort II). JIM 9: 142/964 per arm (Cohort I/Cohort II)

To try to compare the different studies as much as possible, a selection of some of the results reported (in terms of treatment effect, MRI acquisitions and SC level measured) was made—results were selected for 3 T when also data at 1.5 T were available. Notice how power calculations differ considering relapsing-remitting multiple sclerosis/secondary progressive multiple sclerosis and primary progressive multiple sclerosis cohorts.

CIS, clinically isolated syndrome; CSA, cross-sectional area; SCV, spinal cord volume; SCT, Spinal Cord Toolbox; PSIR, phase-sensitive inversion recovery; T1w, T1-weighted; T2w, T2-weighted.

### Intra- and inter-subject variability

There is considerable variability in spinal cord morphology among individuals, with inter-subject cervical CSA standard deviation ∼9%,^86,87,93^ a sizable figure relative to the anticipated multiple sclerosis atrophy rate of ∼2%/year. Evaluating atrophy over time requires repeated MRI scans, which are prone to scan–rescan variability (subject repositioning, motion artefacts and noise), and calculating CSA at each time point poses challenges with analysis pipeline reproducibility, particularly during image segmentation.^90^ Accumulated errors from longitudinal data acquisition and analysis impair the detection of subtle atrophy rates; however, calculating atrophy after co-registering data using a generalized boundary shift integral method can minimize this issue^82^ and semi-automatic CSA segmentation methods^88^; however, the extent to which the aforementioned error accumulation is circumvented depends on the co-registration quality, underscoring the importance of evaluating the sensitivity of cutting-edge methods for measuring atrophy rates.

### Normalization methods

As CSA is an important emerging multiple sclerosis biomarker, minimizing inter-subject variability is crucial for detecting subtle atrophy at a given statistical power.^87^ Various biological markers have been assessed as explanatory variables to reduce CSA variability. The relationship between CSA and age remains inconclusive, despite reports of smaller cord areas in older individuals.^86^ Weight and neck length were also evaluated.^86,94,95^ Currently, there is no universally agreed-upon method for normalizing CSA and GM area,^86^ although total intracranial volume and spinal canal area seem promising normalization metrics.^86,93,96^

Another source of variability is that, typically, CSA is computed at a given vertebral reference; however, the spinal and vertebral level’s correspondence varies across individuals by age and height, as well as within individuals by head tilt. CSA variability is mitigated by using a CNS anatomical reference other than the spine. For example, computing CSA at a certain distance from the pontomedullary junction shows lower intra-subject variability compared with a vertebral-based method.^94,95^

### Acquisition methods

Images require good cord/CSF contrast for CSA computation and good GM/WM contrast for reliable GM tissue assessment. For 3D acquisitions, isotropic voxels are preferred over anisotropic to minimize partial volume bias arising from spinal cord curvature with respect to the imaging slice. However, the long scan times for obtaining high-resolution isotropic data may be prohibitive, and having higher resolution in the axial plane orthogonal to the cord axis is ideal. 2D data are commonly acquired axially, with slices orthogonal to the cord, and in-plane resolution as high as 0.5 × 0.5 mm^2^ and moderate slice thickness ∼3–5 mm to increase signal-to-noise ratio while minimizing partial volume effects due to spine curvature. The preferred choice between isotropic voxels (say 1 mm isotropic) compared with higher axial in-plane (compared with 0.5 mm × 0.5 mm × 4 mm) depends on the degree to which the axial slices can be oriented perpendicular to the cord axis which in turn is dependent on the individual spine curvature and more generally which regions of the spine are included in the field of view. 3D acquisitions are generally more efficient for signal-to-noise ratio per unit acquisition time while shorter 2D acquisitions can be more robust to motion artefacts and partial volume effects for smaller trans-axial field of views. The choice of 2D versus 3D, therefore, also depends on scan time restrictions and coverage needs.

The cord is not always perfectly orthogonal to the slice, which increases partial volume and CSA/GM area bias, and requires corrections to the computed area to account for partial volume and slice angulation with respect to the main axis of the spinal cord. Artefacts can arise from motion, sensitivity to shimming (spin echo preferred over gradient echo to overcome signal dropout biasing segmentation) and ghosting affecting the spinal cord area. The 3D T2-weighted fast spin echo is robust to motion and susceptibility artefacts and provides excellent cord/CSF contrast-to-noise ratio, with a resolution of 0.8 mm isotropic at 3 T using product coils. 3D-Magnetization-Prepared Rapid Gradient Echo/inversion recovery spoiled gradient recalled echo is another popular pulse sequence.^47,97,98^ CSA estimates depend on spinal cord delineation, which depends on relaxation parameters (hence sequence choice of repetition time, echo time, flip angle, etc.).^87^

Protocols for separation of cervical and lumbar GM and WM have been successfully applied in large cohorts of healthy volunteers, amyotrophic lateral sclerosis and multiple sclerosis.^53,86,93,96,99-104^ These methods are based on 2D T2*-weighted sequences (often multi-echo: Multi-Echo Data Image Combination; MEDIC, multi-echo fast field echo; mFFE, MERGE) and strongly T1-weighted 2D protocols (single-slice PSIR, radially sampled averaged magnetization inversion recovery acquisition).^53,86,93,96,98,104^ Different MRI acquisition protocols to assess spinal cord atrophy for the three main vendors (Siemens®, GE® and Philips®) have been proposed.^98,99^ The field’s most complete work, the so-called spine-generic protocol, included protocols to assess atrophy and advanced quantitative MRI methods, involved 42 centres worldwide and resulted in an open-access quantitative MRI database (*n* = 260).^99,105^ The spine-generic protocol is currently being used in a 6-year longitudinal study of 300 people with multiple sclerosis where spinal cord atrophy is one of the study biomarkers.^106^

While the results from research studies suggest that spinal cord atrophy is a very important biomarker for clinical management, the various challenges discussed above need to be met before spinal cord atrophy can provide the necessary precision required for single subject inference. Additional examples include confounding effects of such as pseudo-atrophy similar to that seen in the brain with interferon therapy.^107^ However, the outlook is promising with the recent advancements in longitudinal processing and better understanding of the acquisition methods. For cohort studies and clinical trials, current acquisition strategies and atrophy estimation methods are sufficiently powered.

Given the vast availability of brain MRI compared with more limited dedicated spinal cord MRI, some researchers are assessing atrophy found on the brain MRI in the lower brainstem and what is imaged of the upper cervical spinal cord. Since brain acquisitions are very commonly sagittal, it is possible to increase their field of view in the head/foot direction to include a few vertebral levels of the upper cervical cord, without increasing the acquisition time. When dedicated spinal cord acquisitions are not available, these brain acquisitions extended to the upper cervical cord can be used to reliably assess CSA.^47^ To be able to do so, however, it is fundamental to correctly deal with gradient non-linearity distortions.^97^ Dedicated spinal cord imaging is preferable, and certainly brain MRI does not satisfactorily image the majority of the cervical spinal cord and none of the thoracic spinal cord.^108-110^

## Advanced spinal cord imaging techniques

**Figure 4:**
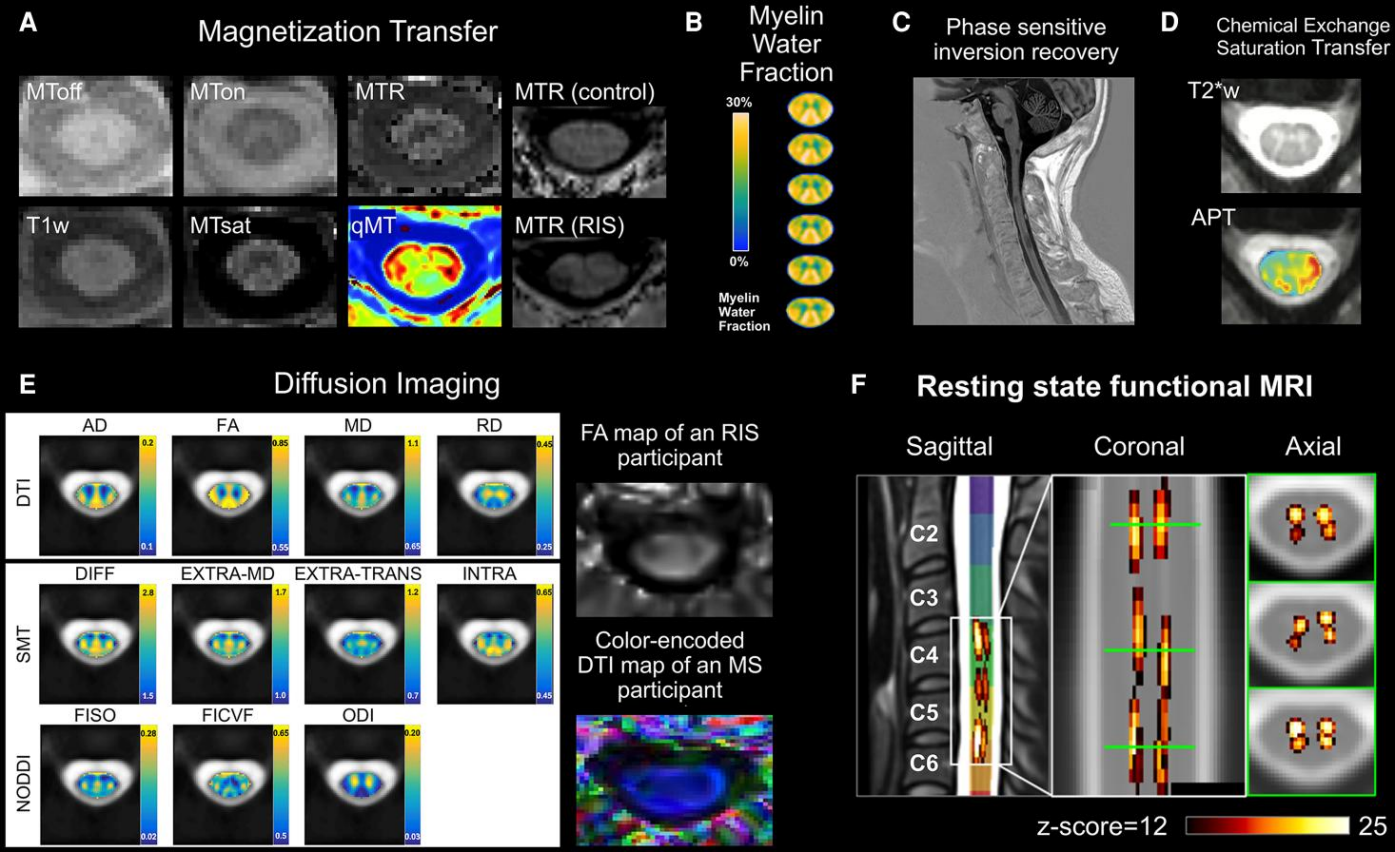
**Advanced imaging techniques in the spinal cord.** (**A**) MT imaging can involve a scan with (MTon) and without (MToff) an off-resonance pulse that is applied to partially saturate the macromolecular pool prior to imaging; the normalized ratio (MToff–MTon/MToff) provides the MTR. A representative cervical cord axial MTR map in an RIS participant shows a decrease in MTR within a wedge-shaped lesion in the right ventral spinal cord relative to a matched control. A T_1_-weighted (T1w) image can be used to calculate MT saturation, which reduces T_1_ dependence to increase inter-scanner agreement. Quantitative MT aims to measure properties of the bound pool. (**B**) Myelin water fraction imaging isolates signal from water between the myelin bilayers based on T_2_ relaxation times. (**C**) PSIR helps highlight lesions; it should be noted that this sagittal protocol is based on a sequence different from the axial technique that has been shown to be important for area quantification and is based on a real reconstruction of a spin-echo inversion recovery sequence. (**D**) Chemical exchange saturation transfer indirectly detects amide protons associated with proteins and peptides quantified by amide proton transfer maps. Many different mathematical and biophysical models exist to extract diffusion-related metrics and microstructural features including (**E**) diffusion tensor imaging, spherical mean technique and neurite orientation dispersion and density imaging to produce metrics such as axial diffusivity, FA, mean diffusivity, radial diffusivity, diffusion coefficient of the isotropic compartment, extra-neurite microscopic mean diffusivity, extra-neurite transverse microscopic diffusivity, intra-cellular volume fraction, isotropic compartment fraction, intracellular volume fraction and orientation dispersion index. (**F**) Resting-state functional MRI demonstrates local alterations in connectivity, which sometimes correlates with diffusion microstructural metrics, suggesting increased connectivity may represent compensatory mechanisms in response to structural damage. Quantitative MT in **A**, **F** in Smith *et al.*,^111^ used with permission. Diffusion imaging in **E** modified from fig. 6 in Schilling *et al.*,^112^ used with permission. AD, axial diffusivity; DIFF, diffusion coefficient of the isotropic compartment; DTA, diffusion tensor imaging; EXTRA-MD, extra-neurite microscopic mean diffusivity; EXTRA-TRANS, extra-neurite transverse microscopic diffusivity; FA, fractional anisotropy; FICVF, intracellular volume fraction; FISO, isotropic compartment fraction; INTRA, intra-cellular volume fraction; MD, mean diffusivity; ODI, orientation dispersion index; qMT, quantitative MT; RD, radial diffusivity.

While conventional MRI methods can provide high-contrast images of the spinal cord that permit lesion identification and assessment of volume and atrophy, these types of clinical scans offer limited specificity and sensitivity to the many pathological processes that can occur in multiple sclerosis. To tackle this challenge, numerous advanced imaging techniques have been developed over the last three decades to probe different aspects of spinal cord structure and function (Fig. 4).

Magnetization transfer (MT) imaging and variants, thereof, including MT saturation, quantitative MT and inhomogeneous MT, are widely used in spinal cord research studies. MT probes magnetization exchange between macromolecular protons and protons in free water, where the MT ratio (MTR) quantifies changes in either proton pool.^113,114^ MT saturation combines MT with T_1_-weighted imaging, which enhances the contrast between tissues with different MT effects and T_1_ relaxation times. Quantitative MT measures bound pool properties, including size, exchange rate and relaxation times.^115^ Finally, inhomogeneous MT is an emerging method that isolates the MT signal dependence on dipolar order relaxation times within motion-constrained molecules, leading to proposed greater myelin lipid specificity.^116^ Evidence of spinal cord microstructural damage in multiple sclerosis is well documented in the MT literature, both *in vivo* and post-mortem.^113,117^ Cross-sectional analysis demonstrates reduced relapsing-remitting multiple sclerosis cervical cord MTR and inhomogeneous MTR relative to matched controls,^92,118,119^ and that MTR abnormalities can be both focal and diffuse.^118^ Longitudinal monitoring reveals reductions in cervical cord MTR at 2 and 5 years, as well as correlations between subject-specific MTR change and EDSS.^34^ Strong correlations between 2- and 5-year cervical cord MTR suggest that changes at 2 years may predict changes at 5 years.^34^ MTR and inhomogeneous MTR cervical cord normal-appearing WM *z*-scores also correlate with disability measures.^119^

Another common advanced imaging technique to study multiple sclerosis spinal cord is diffusion MRI, which probes water movement within tissue in different directions and compartments. Many different mathematical and biophysical models exist to extract different diffusion-related metrics and microstructural features from diffusion data, including diffusion tensor imaging, diffusion kurtosis imaging, neurite orientation dispersion and density imaging, diffusion basis spectrum imaging, q-space imaging and spherical mean technique.^120-122^ Like MT, diffusion is widely used in multiple sclerosis research studies to demonstrate spinal cord microstructural abnormalities.^117,120^ Cervical cord fractional anisotropy (FA) is consistently reduced, and diffusivity measures are elevated in multiple sclerosis, as well as in CIS who converted to clinically definite multiple sclerosis.^92,123-125^ FA of the lateral funiculi can be a predictor of EDSS,^53^ and mean cord FA correlates with nine-hole peg test in relapsing-remitting multiple sclerosis.^124^ Lower FA also significantly correlates with longer latencies measured on the tibialis anterior muscle.^126^ Reduced cervical cord neurite orientation dispersion and density imaging-derived neurite density index correlates with increased EDSS,^127^ and spherical mean technique-derived apparent axonal volume fraction is reduced in lesions and normal-appearing WM compared with controls.^128^ Finally, increased q-space imaging-derived perpendicular diffusivity is associated with disability worsening, where higher cervical cord perpendicular diffusivity (and lower CSA) at baseline predicts worse disability at 3 years.^129^

Numerous other advanced imaging methods are emerging that show promise for studying multiple sclerosis spinal cord pathology. Resting-state functional MRI demonstrates lesions are associated with local alterations in connectivity,^130^ and cervical cord dorsal network functional connectivity correlates with FA, radial and mean diffusivity, suggesting increased connectivity may represent compensatory mechanisms in response to structural damage.^123^ Myelin water imaging reveals cervical cord abnormalities in relapsing-remitting multiple sclerosis and progressive multiple sclerosis and associations with EDSS and nine-hole peg test in progressive patients.^131,132^ The creation of normative myelin water fraction atlases can be used to highlight cervical cord myelin abnormalities in individual patients.^133^ Quantitative susceptibility mapping has the potential to inform on both myelin and iron by quantifying the relative susceptibility between tissues; however, this method has not yet been applied to multiple sclerosis spinal cord^113,134^ Amide proton transfer chemical exchange saturation transfer, which indirectly detects amide protons associated with proteins and peptides, shows multiple sclerosis cord normal-appearing WM amide proton transfer signals are significantly different from controls.^135^ Magnetic resonance spectroscopy quantifies metabolites, including n-acetyl-aspartate, related to neuronal integrity and mitochondrial function. Studies have shown reduced spinal cord n-acetyl-aspartate, as well as correlations between cord n-acetyl-aspartate and EDSS.^113,136^ Cervical cord n-acetyl-aspartate/creatine can also differentiate between focal and diffuse lesions and is significantly associated with cord atrophy, as well as disability progression.^137^ Sodium imaging reports increases in relapsing-remitting multiple sclerosis spinal cord total sodium concentration, which correlates with reduced FA and decreased mediolateral stability.^138^ Improvements in positron emission tomography (PET) hardware, reconstruction and post-processing raises the possibility of quantitative PET imaging in multiple sclerosis spinal cord, but no studies are reported to date.^139^

However, despite the availability of many advanced imaging techniques that can provide specific and quantitative information about multiple sclerosis microstructure, several obstacles hinder their widespread translation to clinical practice, particularly for spinal cord imaging. Technical challenges include artefacts caused by cardiac and respiratory motion, swallowing and CSF pulsations, and the inherently low signal to noise and poor imaging resolution due to the spinal cord’s ability to move and small size—comparable with one’s smallest finger. Harmonization of data acquisition and analysis across different sites and vendors is essential but not straightforward, and specialized pulse sequences may not be available on all systems. In some instances, data acquisition and analysis may also be time-consuming, limiting the feasibility for patient studies. Finally, to identify abnormalities, data from multiple sclerosis must be compared to healthy controls. The relatively small number of normative atlases limits wide-scale implementation. These challenges both demonstrate the critical need and present an opportunity, for continued spinal cord imaging research, with particular emphasis on robustness and ease of implementation for clinical translation.

## Conclusion

Growing evidence increasingly confirms the importance of spinal cord involvement in multiple sclerosis, supporting the need to further advance clinical and research use of spinal cord imaging to investigate multiple sclerosis. The North American Imaging in Multiple Sclerosis provides a structural framework by experts from a variety of institutions combining distinct strengths. Collaborative research by the North American Imaging in Multiple Sclerosis, and similar groups, is key to advancing assessment and treatment of spinal cord disease and ultimately improving outcomes for people living with multiple sclerosis.

## Supplementary Material

fcae395_Supplementary_Data

## Data Availability

Data sharing is not applicable to this article as no new data were created or analysed in this study.
