# Supplementary material for: Spinal cord evaluation in multiple sclerosis: clinical and radiological associations, present and future
Source: Brain Commun. 2024 Nov 6;6(6):fcae395. doi: 10.1093/braincomms/fcae395 (PMC11604059; doi:10.1093/braincomms/fcae395)
Supplement: fcae395_Supplementary_Data [file fcae395_supplementary_data.docx]

Supplementary Table 1 Differential Diagnosis of Myelopathies

| **Onset to nadir,** *disease category,* specific diagnosis^a^ | **Demographic clues** | **Clinical clues** | **Sagittal T2-lesion length & location** | **Axial T2 location** | **T1-Gad+ presence & pattern** | **T1-Gad+ duration** | **Other Spine MRI features** | **Additional useful MRI sequences** | **MRI Brain** | **Other useful diagnostic investigations** |
| --- | --- | --- | --- | --- | --- | --- | --- | --- | --- | --- |
| **Hyper-acute (<12 hrs)** |  |  |  |  |  |  |  |  |  |  |
| *Vascular* (Infarct) | Older adults | Vascular risk factors, trauma/valsalva (dissection, FCE), dorsal column function spared | Usually long; anterior in cord ‘pencil sign’ | Anterior horn cells ‘owl/snake eye’ | Linear anterior strip | A few weeks | May be normal early on | DWI/ADC,  MRA, GRE for blood | Normal or chronic ischemic/non specific white matter T2 hyperintensities | Work-up for mechanism (TEE, holter, thrombphilia panel, lipids, HbA1C) |
| *Functional neurologic disorder* | Young adults, females | Psychiatric disease, stressor, variable exam | None | None | None | N/A | N/A | N/A | Normal | N/A  re-image if further decline or no improvement after months |
| **Acute/Subacute (12 hrs to 21 days)** |  |  |  |  |  |  |  |  |  |  |
| *Immune-mediated with demyelinatiion* |  |  |  |  |  |  |  |  |  |  |
| AQP4+NMOSD | Females, African-Americans, Asian, Hispanic | Area postrema syndrome, optic neuritis, lhermitte’s, tonic spasms | Long (85%), short (15%), | Central, diffuse | Common & 1/3 elongated ring | <3 months | Bright spotty T2 | N/A | Peri-3^rd^ & 4^th^ ventricle, internal capsule, splenium of corps callosum, linear ependymal enhancement | AQP4-IgG serology by cell-based assay |
| MOGAD | Children and adults | ADEM, optic neuritis, Lhermitte’s, | Long (70%), short 30%,  Conus | Central, H-sign | Variable and faint, may have LM Gad+ | <3 months | T2-lesions resolve in follow up | N/A | Ill defined white & deep gray matter, diffuse pons, large MCP, LM Gad+ | MOG-IgG serology by cell-based assay |
| MS | Young adults, Females, | Sensory predominant, mild, lhermitte’s | Short (>99%) | Dorsal, lateral column | Common & 1/3 ring | <3 months | N/A | N/A | T2-lesions in juxtacortical/cortical, periventricular, & inratentorial regions, chronic T1-hypointensity | CSF oligoclonal bands |
| *Infectious*  *(*eg, AFM, schistosomiasis, West Nile Virus*)* | Endemic region, outbreak | Flaccid areflexia with no sensory involvement for AFM | Variable, conus for schistosomiasis | Anterior horn cells for AFM & West Nile | Variable | Variable | Cauda equina | N/A | Normal | Schistosoma (Stool/urine ova, serology, eosinophilia), Enterovirus (nasal swab), West Nile Virus (serology) |
| **Acute/subacute or progressive (12 hrs to months)** |  |  |  |  |  |  |  |  |  |  |
| *Immune-mediated other* |  |  |  |  |  |  |  |  |  |  |
| Paraneoplastic | Older adults | Cancer history | Long | Lateral>dorsal column | Tract-specific | >3 months | May be normal | N/A | Normal | Serum, CSF antibodies (CRMP5, amphiphysin), PET/CT body |
| Other neural antibody-associated | Females | DM1, vitiligo, encehalomyelitis, stiff-person | None (MRI negative myelopathy) | None | None | N/A | None | N/A | Normal | Serum & CSF GAD 65 & Glycine receptor antibodies |
| Rheum/CTD-associated (eg, Sjogren’s) | Variable | Connective tissue disease features | Long | Variable | Variable | Variable | Variable | Normal | None | Rheumatologic markers to include SSA, SSB, include AQP4 and MOG due to high rate of co-occcurence |
| Sarcoid | Variable | Systemic features of sarcoid | Long>short | Central or diffuse | Linear dorsal subpial, axial trident | >3 months | Enlarged hilar nodes visible on T-spine MRI | N/A | Basilar LM enhancement | CT chest, PET/CT, Gallium, biopsy (bronchial, node or nervous system) with non-caseating granulomas, serum ACE and calcium |
| **Progressive (21 days to many months)** |  |  |  |  |  |  |  |  |  |  |
| *Hereditary* (eg, HSP) | Children, young adults | Family history, spasticity predominant | None | None | None | N/A | Diffuse spinal cord atrophy | N/A | Normal or diffuse white matter changes | Genetic testing |
| *Infectious* (eg, HIV, HTLV1, Syphilis) | High-risk behavior, endemic region | Symmetric | Long | Dorsal & lateral columns | Uncommon | Unknown | Ofen MRI is normal | N/A | Normal | Serum HIV, syphilis or HTLV1 serology, CSF VDRL^b^ |
| *Inflammatory demyelinating* (eg, PPMS, progressive solitary sclerosis) | Older adults | Asymmetric myelopathy | Short | Dorsal, lateral column | Very rare | <3 months | Focal atrophic lesions | N/A | T2-lesions in juxtacortical/cortical, periventricular, & inratentorial regions, chronic T1-hypointensity | CSF oligoclonal bands |
| *Neurodegenerative* (eg, amyotrophic lateral sclerosis) | Older adults, family history | Family history, lower motor neuron findings | None | None | None | N/A | N/A | N/A | Normal | EMG, genetic testing |
| *Structural* (eg, spondylosis) | Older adults | Reverse lhermittes, lower motor neuron in upper extremity | Long or short | Central | In 7%, flat pancake-like band, width≥height, axial WM gad+ | >3 months to a few years | Spondylosis, canal stenosis, gad+ slowly resolves post-surgery | Flexion/extension views | Normal | CT myelogram |
| *Metabolic* (eg, B12/N2O, copper, folate); | Variable | Nitrous oxide use, gastric bypass, zinc use | Long | Dorsal>lateral column, rabbit ears | Rare, but can occur (eg, N2O) | Unknown | N/A | N/A | Normal | B12, MMA, copper, folate |
| *Tumor (primary, extrinsic or intramedullary metastases)* | Variable | Known systemic cancer or cancer risk factors | Long or short | Variable | Rim & flame sign for intramedullary metatases | >3 months | Cap sign ependymoma, exophytic | N/A | N/A | PET/CT body, CSF cytology, lesion enalrgement with serial imaging |
| *Vascular* (spinal dAVF, cavernoma) | Older males | Worsening with valsalva, exercise or steroids | Long for DAVF, Thoracic cord, short pop-corn appearance for cavernoma | Central | 50-60%, missing piece of enhancement | >3 months to years | Dorsal flow voids, popcorn appearance for DAVF | MRA to localize dAVF, GRE for blood | Normal | Spinal angiogram to confirm dAVF |

*Abbreviations:* ADEM, acute disseminated encephalomyelitis; AFM, acute flaccid myelitis; AQP4-IgG, aquaporin-4 antibody; AQP4+NMOSD, aquaporin-4 antibody positive neuromyelitis optica spectrum disorder; CRMP5, collapsin response mediator protein 5; CSF, cerebrospinal fluid; CT, computed tomography; CTD, connective tissue disease; dAVF, dural arteriovenous fistula; DM1, type 1 diabetes; DWI/ADC, diffusion weighted imaging/apparent diffusion coefficent; FCE, fibrocartilaginous embolism; GAD 65, glutamic acid decarboxylase 65; GRE, gradient echo; HbA1C, hemoglobin A1C; HIV, human immunodeficiency virus; HSP, hereditary spastic paraplegia; HTLV-1, human T-lymphotropic virus type 1; LM, leptomeningeal; mets, metastases; MCP, middle cerebellar peduncle; MMA, methylmalonic acid; MOG-IgG, Myelin oligodendrocyte glycoprotein antibody; MOGAD, myelin oligodendrocyte glycoprotein antibody-associated disease; MRA, magnetic resonance angiography; MRI, magnetic resonance imaging; MS, multiple sclerosis; N2O, nitrous oxide; neuro, neurologic; PET, positron emission tomography; PPMS, primary progressive multiple sclerosis; T1-Gad+, T1-weighted images post gadolinium; TEE, transesophageal echocardiogram; VDRL, venereal disease research laboratory; wm, white matter.

^a^Time from onset to nadir (bold and underlined), disease category (italics), specific disease (regular text)

^b^Serologic tests (syphilis, HTLV) can be false positive immediately after IVIg treatment, and may need to be confirmed 3 months later.
